# A qualitative investigation of non-response in NHS health checks

**DOI:** 10.1186/s13690-015-0064-1

**Published:** 2015-03-30

**Authors:** Naomi Ellis, Christopher Gidlow, Lisa Cowap, Jason Randall, Zafar Iqbal, Jagdish Kumar

**Affiliations:** Centre for Research in Sport, Health and Exercise, Staffordshire University, Leek Road Campus, Stoke-on-Trent, ST4 2DF UK; Stoke-on-Trent City Council Public Health Directorate, Civic Centre, Glebe Street, Stoke-on-Trent, ST4 1HH UK; Havering Public Health, Mercury House, 12th Floor, Mercury Gardens, Romford, Essex RM1 3DW UK

**Keywords:** Health check, Mass screening, Cardiovascular diseases, Qualitative research

## Abstract

**Background:**

Improving uptake of NHS Health Checks has become a priority in England, but there is a lack of data on the perceptions of programme non-attenders. This study aimed to explore how non-attenders of NHS Health Checks perceive the programme, identify reasons for non-attendance and inform strategies to improve uptake.

**Method:**

This qualitative study involved individuals registered at four general practices in Stoke-on-Trent, UK, who had not taken up their invitation to a NHS Health Check. Semi-structured face-to-face and telephone interviews were audio-recorded and transcribed verbatim for Thematic Analysis.

**Results:**

Interviews were completed with 19 males and 22 females (mean age 52.9 ± 8.5 years), who were socio-demographically representative of the non-attender population. Four main themes identified related to: the positive perception of the Health Check concept among non-attenders; the perceived lack of personal relevance; ineffective invitation method and appointment inconvenience were common barriers; previous experience of primary care can influence uptake.

**Conclusions:**

Fundamental requirements for improving uptake are that individuals recognise the personal relevance of Health Checks and that attendance is convenient. Incorporating more sophisticated and personalised risk communication as part of the invitation could increase impact and promote candidacy. Flexibility and convenience of appointments should be considered by participating general practices.

## Background

The NHS Health Check programme was launched in 2009 in England as a national cardiovascular risk assessment programme [1]. All English adults aged 40 to 74 without a diagnosed chronic condition should be invited for a Health Check. These are typically completed in primary care by Practice Nurses, Health Care Support Workers or General Practitioners, with subsequent treatment, monitoring or signposting to local services to manage any cardiovascular disease (CVD) or associated risk identified. Although some programmes use alternative community venues, such as pharmacies, most Health Checks are delivered in general practice. While debate around effectiveness and cost effectiveness of Health Checks for reducing CVD risk factors and related mortality continues [2-5], a major challenge for such programmes is the rate and equitability of uptake [6,7]; their ability to reach sufficient numbers of the target population to allow population level impact and viability.

A recent national estimate of NHS Health Check uptake of 49% [8] reiterated the need for improvement. Published evidence from NHS Health Checks in England is also indicative of bias in uptake, which tends to be lower in certain groups, such as men, those at the younger end of the target age range and with better health profiles, although patterns by deprivation are less consistent [9-13]. Quantitative process data link higher uptake with verbal methods of Health Check invitation (compared with using only postal invitations) [13] and with smaller practices [14], perhaps because they are better able to engage and provide continuity of care [12].

To date, qualitative data from those who have successfully been engaged by CVD outreach programmes represent the main source of published information about potential shortcomings of recruitment to traditional practice-based Health Checks. Data from interviews with 30 health check non-attenders who were successfully engaged through the Keep Well outreach programme identified reasons for non-attendance including the letter of invitation not having impact, other commitments, and personal beliefs about themselves or the health service [15]. Similarly, focus groups with 13 adults also engaged by outreach work within Have a Heart in Paisley (HaHP) [16], noted issues relating to the process of engagement (e.g., ineffectiveness of postal invitations), lack of understanding of CHD risk (e.g., perceptions of personal good health), service design (e.g., barrier of making the appointment, need for flexibility, preference for non-clinical settings), and lack of priority afforded to the appointment. This accords with findings from a more opportunistic, community health assessment programme [17].

At the time of this study, there were no published qualitative data from non-attenders of NHS Health Checks. Subsequently, there has been a qualitative study of 27 Health Check invitees in South London, 10 of whom were non-attenders [18]. The data from both groups identified challenges including a lack of public awareness of Health Checks, beliefs about personal CVD risk, civic responsibility, issues with appointments and the perceived consequences of a Health Check. We report qualitative data from 41 adults in Stoke-on-Trent, UK, who had not taken up the NHS Health Check invitation (non-attenders) and were not participants of any outreach activity. A qualitative approach was appropriate to provide a deeper understanding of individuals’ perspectives on the Health Check programme, possible explanations for their lack of engagement, and to inform strategies to improve uptake. Data presented complement quantitative data on Health Check uptake in the city [11,13].

## Method

### Settings and participants

Five general practices in Stoke-on-Trent, UK, were recruited, but participants were successfully recruited from four. All participants were individuals who had been sent an invitation to attend a Health Check, but not attended.

### Participant selection and recruitment

To identify non-attenders, general practices distributed invitation letters and participant information sheets on behalf of the evaluation team. Practices were asked to run database searches to identify the last 100 non-attenders to receive letters. Following a low response to the first 500 letters, each practice (where numbers permitted) was asked to distribute letters to the next 100 non-attenders. Letters had tear off reply slips on which individuals were asked to provide a contact telephone number and indicate their consent to be contacted, with a freepost return envelope. A researcher followed up respondents to complete interviews. These were incentivised (£15 per participant), semi-structured, one-to-one interviews that were completed over the telephone (n = 34) or at the general practice (n = 7) at times to suit the participant. This flexibility and use of an incentive were necessary as, by definition, the target group had proved difficult to engage around Health Checks.

### Interview procedures

Interviews were semi-structured, using a list of guide questions. Questions were developed by the researchers, with input from the local Public Health team and two local GPs (Table 1). Questions were reviewed following the first five interviews, with minor amendments to ordering and phrasing. As there were no changes to question content, all interviews were considered comparable and included in analysis. These provided a loose structure to the discussion, whilst allowing the necessary flexibility and freedom for discussions to evolve and be directed by participants. Guide questions made no assumptions with regard to participant perception of Health Checks and their ‘non-attender’ status, and were designed to allow free discussion around the subject. For those with little or no knowledge of Health Checks, or who did not recall being invited, the programme was briefly described and their perceptions about the concept of Health Checks were sought in line with the original guide questions (Table 1). All discussions were audio-recorded and transcribed *verbatim* for analysis.

**Table 1:** **Interview topic guide for NHS Health Check non-attenders, Stoke-on-Trent, 2014**

	**Topic**
1.	Familiarity / knowledge of Health Check Programme
2.	Recall of invitation to attend a Health Check
3.	Impressions of invitation / letter
4.	Feelings about the idea of attending the GP surgery for a Health Check
5.	Any advantages / benefits of attending a Health Check
6.	Any disadvantages of attending a Health Check
7.	Anything that might encourage attendance
8.	Alternative settings for Health Check
9.	Friends and family who have been for a Health check
10.	Friends/family perceptions of interviewee attending a Health check
11.	General state of health
12.	General lifestyle
13.	Use of primary care, visitations to GP
14.	Satisfaction with primary care, GP surgery
15.	Any other comments about the Health Check

Quantitative data were also gathered to provide a basic demographic, health and primary care utilisation profile of participants.

*Socio-demographic.* Participants were asked to specify their age and ethnicity, and gender was recorded. Socio-economic position was estimated using self-reported employment status and the quintile of Index of Multiple Deprivation (IMD) 2010 [19] based on participant postcode.

*Other.* Participants were asked: ‘Do you currently have any medical conditions or chronic illness’ (yes/no); ‘When did you last visit your Doctor?’ (five response categories). Individuals’ recollection of receiving a Health Check invitation was also recorded.

### Data analysis

Data analysis was performed by experienced qualitative researchers. Transcripts were analysed using inductive Thematic Analysis [20]. This involved familiarisation with data through extensive reading, generating preliminary codes, identifying and reviewing themes to ensure that they were data driven. The process allowed for the development of themes that reflected participant opinion. All preliminary codes were identified independently by two experienced qualitative researchers (LC, JR) and verified by a third (NE), before agreement of provisional themes and relationships. These were discussed by the group before being finalised. Themes are discussed in turn and illustrated with participant quotations, which are identified by participant age (yr), gender and whether they did/did not recall the Health Check invitation

## Results

### Sample profile

In total, 894 postal invitations were distributed, with 49 responses (5.5%) and 41 productive interviews (4.6%). There were 19 male (46%) and 22 female (54%) interviewees from four participating practices, with a mean age of 52.9 ± 8.5 years. Overall, demographic profiles of interview participants were compared with the total population of non-attenders from which this sample was drawn. They were similar in terms of age (53.9 ± 9.0 vs. 52.5 ± 8.6, t_(1902)_, p = .308) and gender (46.3 vs. 52.4% male; *χ*^2^ = .862_(1)_, p = .353), with non-significant differences in deprivation (*χ*^2^ = 8.46_(4)_, p = .076) (Table 2).

**Table 2:** **Characteristics of NHS Health Check non-attenders who participated in interviews, Stoke-on-Trent, 2014**

**Characteristic**		**n**	**%**
Practice*			
	1	0	0.0
	2	13	31.7
	3	9	22.0
	4	6	14.6
	5	13	31.7
**Gender**			
	Male	19	46.3
	Female	22	53.7
**Ethnicity**			
	White British	38	92.7
	British Pakistani	2	4.9
	British Asian	1	2.4
**Employment status**			
	Full time	20	48.8
	Part time	8	19.5
	Retired	8	19.5
	Long term sick	4	9.8
	Unemployed	1	2.4
**Deprivation quintile**			
	1 (most deprived)	11	26.8
	2	6	14.6
	3	5	12.2
	4	4	9.8
	5 (least deprived)	7	17.1
	Missing	8	19.5

*Practice 1 distributed the first batch of invitation letters, with no response; the relatively small practice size and high level of Health Check uptake (84%) meant that the practice did not send further invitations.

Self-reported data on awareness of Health Checks, primary care use and personal health indicated that approximately one-third of those interviewed did not recall receiving a Health Check invitation (Table 3), one-quarter reported a pre-existing medical condition and over half had visited their GP within the past three months.

**Table 3:** **Summary data on recollection of Health Check Invitation, self-reported health and general practice use from NHS Health Check non-attenders who participated in interviews, Stoke-on-Trent, 2014**

		**n**	**%**
**Last GP visit, in past…**			
	… week	11	26.8
	… month	7	17.1
	… 3 months	7	17.1
	… 6 months	8	19.5
	… 12 months	3	7.3
	… >12 months	5	12.2
**Preexisting medical conditions**			
	Yes	12	29.3
	No	29	70.7
**Awareness/recollection of HC invitation**			
	No knowledge of HC or invitation	14	34.1
	Remembered invitation	27	65.9

### Themes

The four main themes identified were *Health Check concept*, *Personal relevance*, *Perceptions of general practice* and *Practical barriers to attendance*. Each theme (and sub-themes where appropriate) is discussed in turn using participant quotations to illustrate salient points.

### Health Check concept

The first theme relates to non-attenders’ overall impression of the Health Check concept, including those who did not recall their invitation or have prior knowledge of the programme. The majority of male and female participants reported that a Health Check would be worthwhile and recognised the potential benefit: *"I think it's a good thing that you can be called in and just have a once-over, a bit of an MOT isn't it really*?" (P1, male, 51 yrs, did not recall invitation). Health Checks were described as *‘a good preventative measure’* (P25, female, 47 yrs, did recall invitation), but based on further discussion, the perceived role seemed more related to identifying existing health issues and preventing escalation:

*"It's your choice at the end of day whether you attend or not"* (P8, female, 48 yrs, did recall invitation)

There was evidence that Health Checks were considered an opportunity, which individuals felt entitled to accept or not.

*"It's your choice at the end of day whether you attend or not"* (P8, female, 48 yrs, did recall invitation).

Although true, this expression of autonomy could reflect that non-attenders lacked feelings of obligation to attend that might be more common in screening for other diseases, such as cancers, which are perceived as more severe [21,22]. Some reported that *‘it would be silly not to use [this opportunity]’* (P5, male, 72 yrs, did not recall invitation), and yet none of our participants did. So even when individuals appreciated the role of Health Checks in principle, they did not realise the opportunity themselves. This, combined with data presented under the next theme, suggest a role for further efforts to improve understanding of CVD risk and prevention to highlight the personal relevance of Health Checks.

### Personal relevance

While most of our non-attender participants expressed an interest in having a Health Check, the personal relevance was not clear in many cases: *“It didn’t really apply to me at the time”* (P2, female, 53 yrs, did recall letter). There appeared to be two different health-related reasons; either participants considered themselves to be in good health and did not recognise the preventive role of Health Checks; or they regularly attended general practice for existing health problems and considered additional general checks as irrelevant.

*“It's beneficial for those already having problems…but for me I’m fit and active, you should go when you're poorly, not just for the sake of it”* (P46, male, 66 yrs, did recall invitation)

*"If I hadn't of been coming to the Doctors on a regular basis anyway, probably I would have thought more about taking it up, but because I was already in contact on a regular basis, then I didn't".* (P2, female, 53 yrs, did recall invitation)

This perceived lack of relevance manifested in participants affording a low priority to the Health Check invitations, which were put ‘*at the back of the drawer’*, or at the ‘*bottom of the pile’*:

*"I did have about … two or three letters, … and I have got to say, I just kept putting it off and putting off"* (P17, female, 62 yrs, did recall invitation)

Again, data accord with evidence that the target population do not understand CVD risk or prevention sufficiently well to recognise the role of Health Checks [16], and their personal candidacy; i.e., they did not see themselves as a candidate for Health Checks [23]. The lack of understanding was also evinced through a fear of the consequences of identifying health problems. Concern about discovering existing health conditions was recognised by some female participants: ‘*you go for a check and something is discovered… I hear lots of people end up going for so many tests, and worry about their health*’ (P25, female, 47 yrs, did recall invitation). But in all cases this risk was placed in the context of the benefits of disease prevention, which links to the ‘Health Check Concept’ theme. Conversely, one male participant expressed apprehension because of the associated work and financial implications of disease identification, which was given greater credence than the benefit of early detection (and risk of inaction):

*"finding out if I have got any underlying problems … some people get sick pay don't they, you see I don't, so if I was ill I would [only get] the statutory sick pay"* (P15, male, 44 yrs, did recall invitation)

### Perceptions of general practice

The perceived role and experiences of primary care are likely contributors to individuals’ propensity to take up the Health check invitation. Most participants were satisfied with the care at their general practice:

*"He is one of those that when you walk into his surgery, he is not there with a prescription in his hand to whisk you off … he will listen"* (P25, female, 45 yrs, did recall invitation)

Others, more commonly female participants, expressed some dissatisfaction, which could explain a reticence around Health Check attendance.

*"Sometimes I feel that they don't ask enough, they don't examine you enough … they sit there looking at the computer, look at the notes … they write your prescription and you are gone within five minutes"* (P9, female, 55 yrs, did recall invitation)

*“I am not comfortable [with the GP], but the nurses are brilliant, they are really, really good… If I thought I was going to see him, I wouldn’t bother [attending the Health Check]”* (P2, female, 54 yrs, did recall invitation).

This was not limited to GPs and practice nurses, and included some comments about issues with reception staff when trying to make appointments. The possibility of alternative locations and delivery models could provide the flexibility to accommodate such individual- and practice-specific issues (which links with the Practical Barriers theme).

Other comments indicated that many participants tried to minimise visits to their GP, *“I just tend to avoid the Doctors if I can”* (P14, male, 68 yrs, did recall invitation), and would not visit in the absence of a specific health problem: *“It's very rare I am ill, and I have to be really, really, poorly to go to the Doctors*” (P23, female, 43 yrs, did recall invitation). Linking back to themes around the Health Check Concept and Personal Relevance, greater efforts would be required to motivate such individuals to attend a preventive programme such as Health Check.

### Practical barriers to attendance

The following sub-themes relate to factors that are largely beyond the control and interpretation of the participant.

#### Time

Consistent with the screening literature [12,15], time was a common reason for non-attendance. Being too busy, work commitments and a lack of appointment times outside of working hours were often cited:

*"it is just the time to arrange to go in, … I … come to work early and they are shut. They are shut when I go home. Weekends they are not open, so it's just difficult to get there"* (P9, female, 55 yrs, did recall invitation)

Increasing the flexibility of appointments with weekend or evening provision is an obvious, practical solution that could improve uptake, especially among younger, working-age participants who are less likely to attend [9-13].

*"Saturdays would really, really benefit me and I think that would make it more attractive to attend"* (P23, female, 42 yrs, did recall invitation)

#### Location

Participants were divided on preferred location for Health Checks, but convenience was important and included distance from home/work and car parking provision (in addition to appointment times as detailed previously). A location *‘on my doorstep’* (P13, female, 67 yrs, did recall invitation) was preferable. For many, general practice was most convenient, but others identified alternative community locations, such as the ‘*community centre’* or ‘*Mosque’* (P16, male, 49 yrs, did recall invitation). Some participants also liked *‘the anonymity of going to a different location rather than the GP surgery’* (P47, female, 50 yrs, did recall invitation), which is similar to findings from community outreach programmes where the non-clinical setting and perceived informality can foster engagement [16,17].

#### Invitation letter

About one-third of participants had no recollection of receiving a Health Check invitation letter, which in some cases appeared to explain their non-attendance: *"If I had received it I would have taken it up"* (P6, female, 58 yrs, did not recall invitation). It is possible that some letters were not received because of administrative error [23], but this is unlikely to account for such a high proportion of our sample. Rather, as reported elsewhere [15,16], it is likely that the letter did not have sufficient impact to be remembered (in some cases), let alone to prompt action. Data from those who did recall the invitation letter highlighted several issues that could be addressed with relative ease. First, there was a perceived lack of information about what the Health Check entailed: *"I didn't receive that much information, that's why I never really bothered"* (P4, male, 47 yrs, did recall invitation). Provision of information alone is unlikely to solve the problems around uptake [6], but it could improve understanding to mitigate the aforementioned fear of potential consequences and promote the personal relevance. This might be achieved through personalising the letter using information on recipients’ demographic or health profile.

Second, some reported a lack of reminder or follow up communication, which might have prompted a response if the first invitation had been forgotten:

*"It was just that one letter … and then there's no other follow up … A reminder would be a good thing, I mean it might just give you the little urge to sort of phone up and make an appointment"* (P14, male, 67 yrs, did recall invitation)

Third, even within our predominantly White British sample, the language used in the invitation letter was a barrier: *"when she was reading it she didn't … understand it"* (daughter of P24, female, 43 yrs, did not recall invitation). In this case, the participant reported that the general practice was aware of the language requirements and accommodated them during GP consultations, yet this did not extend to the Health Check invitation letters. Practice population ethnic profiles and language needs are another basic consideration to which Health Check invitation methods must be sensitive.

## Discussion

We report qualitative data from individuals who had not taken up their NHS Health Check invitation (nor been engaged through outreach work). Overall, non-attenders perceived the Health Check concept favourably, but saw it as a way to identify and prevent the worsening of existing issues. Many considered themselves to be too healthy or their current primary care visits too frequent (due to existing health problems) to recognise themselves as candidates for Health Checks. Data showed that the invitation letter often had little or no impact. Approximately one-third of non-attenders did not recall receiving the invitation, whereas others reported a lack of information and demonstrated little understanding of the programme. That none of our sample spoke of verbal or telephone invitations also supports this as a valuable means of engagement where postal approaches fail. Greater flexibility through provision of evening and weekend appointments, and further practical measures to maximise convenience and patient experience of the service, also emerged as important considerations.

Data presented augment evidence from quantitative studies of NHS Health Check uptake [9-13] and qualitative research with those engaged in Health Checks or associated outreach activity [15,16,24]. To date, only one other study has reported qualitative data from Health Check non-attenders (n = 10) [18]. Perhaps the most salient point from our study was that recognising the benefit of the programme, in principle, appeared insufficient in the absence of candidacy, a useful concept when considering engagement (or lack of) with preventive health and screening programmes [23,25,26]. Mackenzie et al. [23] considered candidacy more broadly (not just health-related) in the context of outreach for CVD. The authors note that in more typical ‘reactive’ help-seeking behaviour, individuals identify their own candidacy for healthcare (i.e., illness or symptoms) and act (i.e., present themselves to the healthcare system). In the Health Check scenario, general practices identify candidacy based on eligibility criteria (i.e., age, absence of CVD diagnosis), but are reliant on the individuals receiving an invitation to recognise and accept this candidacy. Our data, particularly those presented under the Personal relevance theme, suggest that this may not happen because people consider themselves in good health, or in regular contact with primary care for other conditions.

To foster health-related candidacy among those invited, it is necessary to promote understanding of CVD risk, prevention and the role of Health Checks [16,24] in a personal and meaningful way, a conclusion consistent with the findings of Burgess et al. [18]. A lack of perceived relevance is also consistent with lower uptake in those at the younger end of the target age range [9-13], who are less likely to have developed symptoms that could alert them to the pertinence of an invitation for CVD risk assessment. The concurrent ineffectiveness of the invitation letter implicates this as a means of communicating the personal relevance of Health Checks. This could be through personal tailoring of the invitation based on important factors, such as the individual’s age, and health or CVD risk profile. The need for tailoring has been acknowledged in relation to outreach work [16], and local adaptations of the Health Check letter have been trialled in some localities (e.g., Medway, Bromley). Yet more sophisticated ways to recruit to practice-based Health Checks should be explored, to address the previously noted limitations of postal invitations [15,16]. For example, practitioners and researchers could explore using patient CVD risk to highlight the relevance for prevention or potential treatment, and the use of concepts such as Heart Age [27], to improve communication around CVD risk and the role of Health Checks within the written or verbal invitations. This could also help to address the ‘fear of the consequences’ of a Health Check that were evident in our sample and other published qualitative data relating to health checks [7,18]. We are not the first to highlight that flexibility of appointments and convenience are important practical requirements to address the commonly reported time barrier that can deter attendance of screening [12,15-17]. But these are the first data from a sample comprised solely of Health Check non-attenders to make this point, which, again, could explain the consistently lower attendance among the younger, working-age participants. Data also provide confirmation that the perceived role and previous experiences of primary care are likely to influence Health Check attendance [16,17]. Although our sample had good representation of both male and female non-attenders across the Health Check age range, we did not find consistent gender or age patterns in themes. A recent qualitative study similarly found no evidence of gender differences in emergent themes from attenders and non-attenders of Health Checks [18]. The authors did, however, report that appointment convenience appeared more problematic in the younger, working age groups, compared with older participants. Although intuitive and consistent with patters in uptake, we perhaps did not see this because our sample comprised only non-attenders. The most notable observation linking to participant demographics was that more female than males expressed some dissatisfaction with primary care based on previous experiences, but again, our novel focus on accruing a large non-attender sample might have precluded the possibility of detecting gender-specific issues regarding attendance/non-attendance.

The study has a number of strengths and limitations. We gathered a large qualitative dataset from a population that is difficult to engage with. Our sample size (n = 41) compares well with other qualitative research in the area (n = 30 [15] n = 10 [24]). Although we cannot assume representativeness, quantitative data showed sample socio-demographics similar to the overall non-attender population from these practices and that levels of general primary care use was varied (i.e., participants were not simply non-users of primary care). This confirmed that incentivised telephone and face-to-face interviews enabled us to reach the target group. Moreover, the two interview methods provided data of comparable richness, such that all were treated as a single dataset. Experienced qualitative researchers were involved in all stages of study design, data collection and analysis, providing confidence that themes were a fair reflection of participant responses and that data saturation was reached. We recognise some limitations. First, participants were drawn from a small number of general practices in one city, so generalisability to other areas may be limited. Second, eleven participants self-reported a pre-existing medical condition. For the majority it would not have affected their Health Check eligibility. Four participants declined to expand; therefore we cannot be certain of eligibility or whether their reported condition pre-dated the Health Check invitation, and as a result, data were included. Given that health was cited as a reason for non-attendance by some this is an important consideration.

## Conclusions

Increasing uptake is a national priority for NHS Health Checks. Our data offer valuable feedback from those currently not engaged through usual methods, highlighting two fundamental requirements for improving uptake at practice-based Health Checks: individuals see the personal relevance of Health Checks; appointments are convenient.

The weakness of the standard postal invitation and the need to convey the personal relevance of Health Checks to the whole target group are areas for development. Simple changes to the invitation letter have been linked with modest improvements in attendance elsewhere (29 vs. 33% uptake) [28]. We propose that more sophisticated and personalised risk communication could be tested to increase the impact of the letter, in addition to further exploring use of telephone/verbal methods [13]. Basic measures to maximise convenience include weekend and evening provision, and consideration of the local population and their requirements (e.g., language barriers, parking, ease of making appointments). Ideally, measures that are tested would be part of natural experiments, with sufficient control groups and data collection processes to determine effectiveness.

Finally, common themes that have emerged from other research involving outreach and more opportunistic health assessment programmes, implicate these as important adjuncts to practice-based delivery.

### Ethical approval

The study was approved by the University Ethics committee.
